# Supplementary material for: Facial Appearance and Psychosocial Features in Orthognathic Surgery: A FACE-Q- and 3D Facial Image-Based Comparative Study of Patient-, Clinician-, and Lay-Observer-Reported Outcomes
Source: J Clin Med. 2019 Jun 25;8(6):909. doi: 10.3390/jcm8060909 (PMC6616869; doi:10.3390/jcm8060909)
Supplement: Supplementary file 1 [file jcm-08-00909-s001.zip › Tables S1 to S8.docx]

**Table S1.** Rating Scales Adopted in the Panel Assessment [17-23].

| **Rating scales** | **Scores** | **Descriptions** |
| --- | --- | --- |
| **Facial aesthetic** |  |  |
| Beautiful | 1 to 7 | 1 = ugly; 7 = beautiful |
| Attractive | 1 to 7 | 1 = unattractive; 7 = attractive |
| Pleasant | 1 to 7 | 1 = unpleasant; 7 = pleasant |
| **Facial personality traits** |  |  |
| Intelligent | 1 to 7 | 1 = unintelligent; 7 = intelligent |
| Friendly | 1 to 7 | 1 = unfriendly; 7 = friendly |
| Threatening | 1 to 7 | 1 = nonthreatening; 7 = threatening |
| Trustworthy | 1 to 7 | 1 = untrustworthy; 7 = trustworthy |
| Dominant | 1 to 7 | 1 = submissive; 7 = dominant |
| **Facial emotional expressions** |  |  |
| Angry | 1 to 7 | 1 = very; 7 = no at all |
| Surprised | 1 to 7 | 1 = very; 7 = no at all |
| Happy | 1 to 7 | 1 = very; 7 = no at all |
| Sad | 1 to 7 | 1 = very; 7 = no at all |
| Afraid | 1 to 7 | 1 = very; 7 = no at all |
| Disgusted | 1 to 7 | 1 = very; 7 = no at all |
|  | | |

**Table S2.** Reliability Data for the Panel Assessment Tool Used in this Study.

| **Scales** | **Three groups**  **of raters** | **Orthodontics**  **Versus**  **Surgeons** | **Orthodontics**  **Versus**  **Lay observers** | **Surgeons**  **Versus**  **Lay** **observers** |
| --- | --- | --- | --- | --- |
| **Facial aesthetic** |  |  |  |  |
| Cronbach’s Alpha (*p*)* | 0.79-0.86(<0.01) | – | – | – |
| ICC (intra/inter)** | 0.68-0.95/0.74-0.97 | – | – | – |
| *r* (*p*)† | – | 0.12-0.23 (<0.01) | 0.14-0.22 (<0.01) | 0.02-0.15 (<0.01) |
| **Personality traits** |  |  |  |  |
| Cronbach’s Alpha (*p*)* | 0.75-0.89 (<0.01) | – | – | – |
| ICC (intra/inter)** | 0.62-0.93/0.69-0.94 | – | – | – |
| *r* (*p*)† | – | 0.13-0.16 (<0.01) | 0.11-0.18 (<0.01) | 0.19-0.24 (<0.01) |
| **Emotional expressions** |  |  |  |  |
| Cronbach’s Alpha (*p*)* | 0.72-0.84 (<0.01) | – | – | – |
| ICC (intra/inter)** | 0.65-0.86/0.67-0.91 | – | – | – |
| *r* (*p*)† | – | 0.11-0.24 (<0.01) | 0.13-0.22 (<0.01) | 0.17-0.28 (<0.01) |
| Intra, intra-rater reliability; Inter, inter-rater reliability; ICC, intraclass correlation coefficient for multiple raters; r, correlation coefficient; p, p-value; –, not applicable;  *, Coefficient Cronbach alpha value (> 0.7 = acceptable for internal-consistency of a measurement scale; p < 0.05 mean that the data are internally consistent);  *, ICC was interpreted as fair (0.40 to 0.59), good (0.60 and 0.74), and excellent (0.75 to 1.00);  †, correlation between the groups of raters (p < 0.05 mean that the rater scores are correlated) | | | | |

**Table S3.** Correlations for Pre- and Post-Orthognathic Surgery Assessment Scores of FACE-Q Facial Appraisal Scales and Orthodontic-Based Panel Assessment of Facial Aesthetic Scales.

| **Facial aesthetic**  **scales** | **FACE-Q tool** | | | **Panel assessment tool** | | |
| --- | --- | --- | --- | --- | --- | --- |
|  | **Appearance overall** | **Lower face and jaw** | **Lips** | **Beautiful** | **Attractive** | **Pleasant** |
|  | ***r* (*p*)** | ***r* (*p*)** | ***r* (*p*)** | ***r* (*p*)** | ***r* (*p*)** | ***r* (*p*)** |
| **Beautiful** |  |  |  |  |  |  |
| Pre-OGS | 0.28  (<0.01) | 0.16  (>0.05) | 0.08  (<0.05) | – | – | – |
| Post-OGS | 0.17  (<0.01) | 0.08  (>0.05) | 0.16  (<0.05) | – | – | – |
| **Attractive** |  |  |  |  |  |  |
| Pre-OGS | 0.25  (<0.01) | 0.22  (>0.05) | 0.11  (<0.05) | 0.19  (<0.01) | – | – |
| Post-OGS | 0.21  (<0.01) | 0.13  (>0.05) | 0.18  (<0.05) | 0.15  (<0.01) | – | – |
| **Pleasant** |  |  |  |  |  |  |
| Pre-OGS | 0.06  (<0.01) | 0.17  (>0.05) | 0.14  (>0.05) | 0.13  (<0.01) | 0.16  (<0.01) | – |
| Post-OGS | 0.29  (>0.05) | 0.04  (>0.05) | 0.12  (>0.05) | 0.06  (<0.01) | 0.10  (<0.01) | – |
| OGS, orthognathic surgery; r, correlation coefficient; p, p-value; –, not applicable | | | | | | |

**Table S4.** Correlations for Pre- and Post-Orthognathic Surgery Assessment Scores of FACE-Q Facial Appraisal Scales and Lay Observer-Based Panel Assessment of Facial Aesthetic Scales.

| **Facial aesthetic**  **scales** | **FACE-Q tool** | | | **Panel assessment tool** | | |
| --- | --- | --- | --- | --- | --- | --- |
|  | **Appearance overall** | **Lower face**  **and jaw** | **Lips** | **Beautiful** | **Attractive** | **Pleasant** |
|  | ***r* (*p*)** | ***r* (*p*)** | ***r* (*p*)** | ***r* (*p*)** | ***r* (*p*)** | ***r* (*p*)** |
| **Beautiful** |  |  |  |  |  |  |
| Pre-OGS | 0.12  (<0.01) | 0.17  (>0.05) | 0.08  (<0.05) | – | – | – |
| Post-OGS | 0.04  (<0.01) | 0.07  (>0.05) | 0.29  (<0.05) | – | – | – |
| **Attractive** |  |  |  |  |  |  |
| Pre-OGS | 0.15  (<0.01) | 0.16  (>0.05) | 0.23  (<0.05) | 0.39  (<0.01) | – | – |
| Post-OGS | 0.10  (<0.01) | 0.21  (>0.05) | 0.18  (<0.05) | 0.34  (<0.01) | – | – |
| **Pleasant** |  |  |  |  |  |  |
| Pre-OGS | 0.13  (>0.05) | 0.18  (>0.05) | 0.23  (>0.05) | 0.11  (<0.01) | 0.14  (<0.01) | – |
| Post-OGS | 0.22  (>0.05) | 0.05  (>0.05) | 0.17  (>0.05) | 0.08  (<0.01) | 0.05  (<0.01) | – |
| OGS, orthognathic surgery; r, correlation coefficient; p, p-value; –, not applicable | | | | | | |

**Table S5.** Correlations for Pre- and Post-Orthognathic Surgery Assessment Scores of FACE-Q Quality of Life Scales and Surgeon-Based Panel Assessment of Personality Traits Scales.

| **Personality traits scales** | **FACE-Q tool** | | **Panel assessment tool** | | | | |
| --- | --- | --- | --- | --- | --- | --- | --- |
|  | **Social** | **Psychological** | **Intelligent** | **Friendly** | **Threat** | **Trust** | **Dominant** |
|  | ***r* (*p*)** | ***r* (*p*)** | ***r* (*p*)** | ***r* (*p*)** | ***r* (*p*)** | ***r* (*p*)** | ***r* (*p*)** |
| **Intelligent** |  |  |  |  |  |  |  |
| Pre-OGS | 0.10  (>0.05) | 0.18  (>0.05) | – | – | – | – | – |
| Post-OGS | 0.09  (>0.05) | 0.24  (>0.05) | – | – | – | – | – |
| **Friendly** |  |  |  |  |  |  |  |
| Pre-OGS | 0.05  (>0.05) | 0.16  (>0.05) | 0.22  (>0.05) | – | – | – | – |
| Post-OGS | 0.11  (>0.05) | 0.04  (>0.05) | 0.07  (>0.05) | – | – | – | – |
| **Threat** |  |  |  |  |  |  |  |
| Pre-OGS | 0.17  (>0.05) | 0.34  (>0.05) | 0.08  (>0.05) | 0.13  (>0.05) | – | – | – |
| Post-OGS | 0.09  (>0.05) | 0.15  (>0.05) | 0.18  (>0.05) | 0.02  (>0.05) | – | – | – |
| **Trust** |  |  |  |  |  |  |  |
| Pre-OGS | 0.09  (>0.05) | 0.21  (>0.05) | 0.06  (>0.05) | 0.27  (>0.05) | 0.24  (>0.05) | – | – |
| Post-OGS | 0.19  (>0.05) | 0.14  (>0.05) | 0.12  (>0.05) | 0.19  (>0.05) | 0.15  (>0.05) | – | – |
| **Dominant** |  |  |  |  |  |  |  |
| Pre-OGS | 0.19  (>0.05) | 0.22  (>0.05) | 0.10  (>0.05) | 0.35  (>0.05) | 0.07  (>0.05) | 0.24  (>0.05) | – |
| Post-OGS | 0.06  (>0.05) | 0.17  (>0.05) | 0.26  (>0.05) | 0.02  (>0.05) | 0.13  (>0.05) | 0.05  (>0.05) | – |
| OGS, orthognathic surgery; Threat, threatening; Trust, trustworthy; r, correlation coefficient; p, p-value; –, not applicable | | | | | | | |

**Table S6.** Correlations for Pre- and Post-Orthognathic Surgery Assessment Scores of FACE-Q Quality of Life Scales and Lay Observer-Based Panel Assessment of Personality Traits Scales.

| **Personality traits scales** | **FACE-Q tool** | | **Panel assessment tool** | | | | |
| --- | --- | --- | --- | --- | --- | --- | --- |
|  | **Social** | **Psychological** | **Intelligent** | **Friendly** | **Threat** | **Trust** | **Dominant** |
|  | ***r* (*p*)** | ***r* (*p*)** | ***r* (*p*)** | ***r* (*p*)** | ***r* (*p*)** | ***r* (*p*)** | **r (p)** |
| **Intelligent** |  |  |  |  |  |  |  |
| Pre-OGS | 0.09  (>0.05) | 0.23  (>0.05) | – | – | – | – | – |
| Post-OGS | 0.14  (>0.05) | 0.11  (>0.05) | – | – | – | – | – |
| **Friendly** |  |  |  |  |  |  |  |
| Pre-OGS | 0.13  (>0.05) | 0.36  (>0.05) | 0.15  (>0.05) | – | – | – | – |
| Post-OGS | 0.09  (>0.05) | 0.18  (>0.05) | 0.12  (>0.05) | – | – | – | – |
| **Threat** |  |  |  |  |  |  |  |
| Pre-OGS | 0.11  (>0.05) | 0.16  (>0.05) | 0.24  (>0.05) | 0.10  (>0.05) | – | – | – |
| Post-OGS | 0.17  (>0.05) | 0.04  (>0.05) | 0.19  (>0.05) | 0.22  (>0.05) | – | – | – |
| **Trust** |  |  |  |  |  |  |  |
| Pre-OGS | 0.14  (>0.05) | 0.02  (>0.05) | 0.20  (>0.05) | 0.19  (>0.05) | 0.09  (>0.05) | – | – |
| Post-OGS | 0.27  (>0.05) | 0.09  (>0.05) | 0.14  (>0.05) | 0.03  (>0.05) | 0.38  (>0.05) | – | – |
| **Dominant** |  |  |  |  |  |  |  |
| Pre-OGS | 0.07  (>0.05) | 0.14  (>0.05) | 0.19  (>0.05) | 0.23  (>0.05) | 0.11  (>0.05) | 0.06  (>0.05) | – |
| Post-OGS | 0.19  (>0.05) | 0.30  (>0.05) | 0.08  (>0.05) | 0.24  (>0.05) | 0.04  (>0.05) | 0.13  (>0.05) | – |
| OGS, orthognathic surgery; Threat, threatening; Trust, trustworthy; r, correlation coefficient; p, p-value; –, not applicable | | | | | | | |

**Table S7.** Correlations for Pre- and Post-Orthognathic Surgery Assessment Scores of FACE-Q Quality of Life Scales and Surgeon-Based Panel Assessment of Emotional Expressions Scales.

| **Emotional expressions scales** | **FACE-Q tool** | | **Panel assessment tool** | | | | | |
| --- | --- | --- | --- | --- | --- | --- | --- | --- |
|  | **Social** | **Psychological** | **Angry** | **Surprised** | **Happy** | **Sad** | **Afraid** | **Disgusted** |
|  | ***r* (*p*)** | ***r* (*p*)** | ***r* (*p*)** | ***r* (*p*)** | ***r* (*p*)** | ***r* (*p*)** | ***r* (*p*)** | ***r* (*p*)** |
| **Angry** |  |  |  |  |  |  |  |  |
| Pre-OGS | 0.36  (>0.05) | 0.33  (>0.05) | – | – | – | – | – | – |
| Post-OGS | 0.27  (>0.05) | 0.02  (>0.05) | – | – | – | – | – | – |
| **Surprised** |  |  |  |  |  |  |  |  |
| Pre-OGS | 0.18  (>0.05) | 0.25  (>0.05) | 0.09  (>0.05) | – | – | – | – | – |
| Post-OGS | 0.21  (>0.05) | 0.02  (>0.05) | 0.27  (>0.05) | – | – | – | – | – |
| **Happy** |  |  |  |  |  |  |  |  |
| Pre-OGS | 0.36  (>0.05) | 0.32  (>0.05) | 0.17  (>0.05) | 0.29  (>0.05) | – | – | – | – |
| Post-OGS | 0.09  (>0.05) | 0.07  (>0.05) | 0.12  (>0.05) | 0.30  (>0.05) | – | – | – | – |
| **Sad** |  |  |  |  |  |  |  |  |
| Pre-OGS | 0.03  (>0.05) | 0.17  (>0.05) | 0.19  (>0.05) | 0.01  (>0.05) | 0.26  (>0.05) | – | – | – |
| Post-OGS | 0.17  (>0.05) | 0.15  (>0.05) | 0.08  (>0.05) | 0.32  (>0.05) | 0.14  (>0.05) | – | – | – |
| **Afraid** |  |  |  |  |  |  |  |  |
| Pre-OGS | 0.11  (>0.05) | 0.14  (>0.05) | 0.12  (>0.05) | 0.38  (>0.05) | 0.11  (>0.05) | 0.38  (>0.05) | – | – |
| Post-OGS | 0.32  (>0.05) | 0.04  (>0.05) | 0.12  (>0.05) | 0.25  (>0.05) | 0.23  (>0.05) | 0.16  (>0.05) | – | – |
| **Disgusted** |  |  |  |  |  |  |  |  |
| Pre-OGS | 0.01  (>0.05) | 0.14  (>0.05) | 0.18  (>0.05) | 0.20  (>0.05) | 0.11  (>0.05) | 0.38  (>0.05) | 0.14  (>0.05) | – |
| Post-OGS | 0.08  (>0.05) | 0.36  (>0.05) | 0.22  (>0.05) | 0.34  (>0.05) | 0.19  (>0.05) | 0.26  (>0.05) | 0.21  (>0.05) | – |
| OGS, orthognathic surgery; r, correlation coefficient; p, p-value; –, not applicable | | | | | | | | |

**Table S8.** Correlations for Pre- and Post-Orthognathic Surgery Assessment Scores of FACE-Q Quality of Life Scales and Orthodontic-Based Panel Assessment of Emotional Expressions Scales.

| **Emotional expressions scales** | **FACE-Q tool** | | **Panel assessment tool** | | | | | |
| --- | --- | --- | --- | --- | --- | --- | --- | --- |
|  | **Social** | **Psychological** | **Angry** | **Surprised** | **Happy** | **Sad** | **Afraid** | **Disgusted** |
|  | ***r* (*p*)** | ***r* (*p*)** | ***r* (*p*)** | ***r* (*p*)** | ***r* (*p*)** | ***r* (*p*)** | ***r* (*p*)** | ***r* (*p*)** |
| **Angry** |  |  |  |  |  |  |  |  |
| Pre-OGS | 0.12  (>0.05) | 0.08  (>0.05) | – | – | – | – | – | – |
| Post-OGS | 0.19  (>0.05) | 0.22  (>0.05) | – | – | – | – | – | – |
| **Surprised** |  |  |  |  |  |  |  |  |
| Pre-OGS | 0.12  (>0.05) | 0.06  (>0.05) | 0.05  (>0.05) | – | – | – | – | – |
| Post-OGS | 0.33  (>0.05) | 0.14  (>0.05) | 0.15  (>0.05) | – | – | – | – | – |
| **Happy** |  |  |  |  |  |  |  |  |
| Pre-OGS | 0.27  (>0.05) | 0.17  (>0.05) | 0.13  (>0.05) | 0.22  (>0.05) | – | – | – | – |
| Post-OGS | 0.12  (>0.05) | 0.35  (>0.05) | 0.08  (>0.05) | 0.31  (>0.05) | – | – | – | – |
| **Sad** |  |  |  |  |  |  |  |  |
| Pre-OGS | 0.12  (>0.05) | 0.29  (>0.05) | 0.18  (>0.05) | 0.34  (>0.05) | 0.27  (>0.05) | – | – | – |
| Post-OGS | 0.09  (>0.05) | 0.15  (>0.05) | 0.30  (>0.05) | 0.09  (>0.05) | 0.19  (>0.05) | – | – | – |
| **Afraid** |  |  |  |  |  |  |  |  |
| Pre-OGS | 0.28  (>0.05) | 0.04  (>0.05) | 0.37  (>0.05) | 0.02  (>0.05) | 0.19  (>0.05) | 0.22  (>0.05) | – | – |
| Post-OGS | 0.09  (>0.05) | 0.20  (>0.05) | 0.18  (>0.05) | 0.22  (>0.05) | 0.15  (>0.05) | 0.01  (>0.05) | – | – |
| **Disgusted** |  |  |  |  |  |  |  |  |
| Pre-OGS | 0.31  (>0.05) | 0.16  (>0.05) | 0.27  (>0.05) | 0.18  (>0.05) | 0.12  (>0.05) | 0.36  (>0.05) | 0.18  (>0.05) | – |
| Post-OGS | 0.04  (>0.05) | 0.10  (>0.05) | 0.28  (>0.05) | 0.24  (>0.05) | 0.13  (>0.05) | 0.15  (>0.05) | 0.07  (>0.05) | – |
| OGS, orthognathic surgery; r, correlation coefficient; p, p-value; –, not applicable | | | | | | | | |
